# Genome-wide characterization of ubiquitin-conjugating enzyme gene family explores its genetic effects on the oil content and yield of *Brassica napus*

**DOI:** 10.3389/fpls.2023.1118339

**Published:** 2023-03-20

**Authors:** Shengli Yao, Meili Xie, Ming Hu, XiaoBo Cui, Haoming Wu, Xiaohua Li, Peng Hu, Chaobo Tong, Xiaoli Yu

**Affiliations:** ^1^ School of Life Science and Technology, Wuhan Polytechnic University, Wuhan, Hubei, China; ^2^ The Key Laboratory of Biology and Genetic Improvement of Oil Crops, the Ministry of Agriculture and Rural Affairs of the PRC, Oil Crops Research Institute of the Chinese Academy of Agricultural Sciences, Wuhan, China

**Keywords:** *Brassica napus*, the *UBC* family, evolution, association mapping analysis, oil content and yield

## Abstract

Ubiquitin-conjugating enzyme (UBC) is a critical part of the ubiquitin–proteasome pathway and plays crucial roles in growth, development and abiotic stress response in plants. Although *UBC* genes have been detected in several plant species, characterization of this gene family at the whole-genome level has not been conducted in *Brassica napus*. In the present study, 200 putative *BnUBCs* were identified in *B. napus*, which were clustered into 18 subgroups based on phylogenetic analysis. *BnUBC*s within each subgroup showed relatively conserved gene architectures and motifs. Moreover, the gene expression patterns in various tissues as well as the identification of *cis*-acting regulatory elements in *BnUBC* promoters suggested further investigation of their potential functions in plant growth and development. Furthermore, three *BnUBC*s were predicted as candidate genes for regulating agronomic traits related to oil content and yield through association mapping. In conclusion, this study provided a wealth of information on the *UBC* family in *B. napus* and revealed their effects on oil content and yield, which will aid future functional research and genetic breeding of *B. napus*.

## Introduction

Ubiquitination is a crucial regulatory process for the selective protein degradation mechanism which regulates cell physiology through the ubiquitin-26S proteasome pathway in plants (Pickart, 2001; Smalle and Vierstra, 2004; Vierstra, 2009; Ye and Rape, 2009). Ubiquitination regulates a wide range of various plant growth and developmental processes including photomorphogenesis, protein translocation within cells, flower development, cell cycle control, both abiotic and biotic stress responses, phytohormone, regulation of proteome homeostasis as well as light signaling (Welchman et al., 2005; Dreher and Callis, 2007; Vierstra, 2009; Sadanandom et al., 2012; Doroodian and Hua, 2021; Yu and Hua, 2022). In addition, the ubiquitin-26S proteasome system (UPS) plays critical role in plant adaption (Hua and Yu, 2019). Protein ubiquitination involves the covalent attachment of a 76-amino acid (aa) sequence, through one of the encompassing seven lysine residues (Lys-6, Lys-11, Lys-27, Lys-29, Lys-33, Lys-48, Lys-63), to substrate protein (Welchman et al., 2005; Kim et al., 2013). Moreover, the fate of the ubiquitinated substrate is determined by the type of ubiquitination as well as the choice of the Lys residue for the modification, resulting in different linkages with various functions (Fang and Weissman, 2004; Sun and Chen, 2004).

The protein ubiquitin-proteasome system (UPS) is a multistep reaction mediated by three enzymes, E1 (ubiquitin-activating enzyme, UBA), E2 (ubiquitin-conjugating enzyme, UBC) and E3 (Ubiquitin-ligase enzyme) (Glickman and Ciechanover, 2002). Ubiquitin is initially activated by E1 through the ATP-dependent reaction, which results in the formation of a thioester-linked ubiquitin (Ramadan et al., 2015). Then, E1 transfers the thioester-linked ubiquitin to the cysteine (Cys) residue (active site) of the UBC domain through the passing of E2 *via* transesterification (Hershko et al., 1983). Subsequently, E2 transfers the ubiquitin moiety to the substrate protein with the help of E3 directly *via* a second *ans*-thiolation reaction, which mediates the formation of polyubiquitin chains on target proteins and determines the specificity of the substrate in the ubiquitination system (Bae and Kim, 2013; Bae and Kim, 2014). Finally, the 26S proteasome degrades the target protein. The family of UBCs (E2s) is central to this enzymatic cascade, which offers a platform for the attachment of ubiquitin to the target proteins (Hershko et al., 1983; Burroughs et al., 2008).

Most ubiquitin-like conjugating enzymes (UBLs) and E2s comprise a conserved catalytic core consisting of approximately 140-150 aa known as the UBC domain harboring the active site cysteine residue required for enzyme-ubiquitin (Criqui et al., 2002; Michelle et al., 2009; Schumacher et al., 2013). Moreover, several studies indicated that the UBC domain contributes to the mediation of the interaction between E2 and E3 (Huang et al., 1999; Christensen et al., 2007; Poyurovsky et al., 2007). The UBC domains in various members of the E2 family is highly conserved, both in terms of amino acid sequence and three-dimensional structure, which possessed four α-helices, an anti-parallel β-sheet formed by four strands, and a short 310–helix (Lin et al., 2002; Ozkan et al., 2005; Wenzel et al., 2011). Among the multiple binding sites, one highly conserved active-site (Cys residue) is located at a shallow groove formed by a short loop connecting α-helix 2 with α-helix 3 as well as a proximal long loop (Ye and Rape, 2009; van Wijk and Timmers, 2010). The formation of E2-ubiquitin thioester requires E2s to interact with ubiquitin as well as with E1 (or its cognates) which results in considerable evolutionary constraints on E2 structure and the formation of the conserved active site (Silver et al., 1992). Aside from the core E2 domain, some detected E2 enzymes comprise diverse N- and C-terminal extensions that are thought to contribute to the intracellular localization of the enzyme and its substrate specificity (Arrigoni et al., 2012; Hodson et al., 2014). Furthermore, UBC enzymes are divided into four classes, based on the N- and C-terminal extensions as well as the UBC domain. Class I UBCs contain only the UBC domain with a region of approximately 150 conserved residues, whereas Class II UBCs comprise the N-terminal extension and the UBC domain; Class III E2s harbor the C-terminal extension as well as the UBC domain; and Class IV UBCs possess both the N- and C-terminal extensions and the UBC domain (Papaleo et al., 2012; Schumacher et al., 2013).

The genes encoding *UBCs* usually form a multigene family and the number of *UBC*s in the multigene family is greater in the higher eukaryotes than in the lower eukaryotes, due to the expansion during the process of evolution (Jue et al., 2015). Moreover, UBLs, including the SUMO-conjugating enzymes, ubiquitin (RUB) conjugating enzymes and ubiquitin-conjugating enzyme variants (UEVs) also belong to the E2 category. Among the 48 UBC domain-containing proteins identified in *Arabidopsis* (Kraft et al., 2005), except 37 potential E2s, three are thioester-linked UBLs, one is a SUMO-conjugating enzyme [AtSCE1, At3g57870), and two are RUB-conjugating related enzymes (At4g36800 (RCE1) and At2g18600 (RCE2)]. These UBL-specific enzymes perform the same function as E2s, but they do not belong to the *UBC* family. In addition, eight UBC proteins are termed as ubiquitin-conjugating enzyme variants (UEVs), since they lack the active-site Cys residue, which is not active on their own (Callis, 2014). Based on sequence homology, the 48 *Arabidopsis UBCs* were classified into 16 groups (Kraft et al., 2005). Plant UBC proteins, as previous studies reported, play a crucial role in regulating plant development, growth and stress response (Dreher and Callis, 2007; Sadanandom et al., 2012; E et al., 2015; Millyard et al., 2016; Jue et al., 2018; Kravic et al., 2020; Liu et al., 2020). For example, functional analyses have revealed that *AtUBC1* and *AtUBC2* are involved in leaf development, tolerance response to UV stress, as well as activation of the floral repressor gene (Xu et al., 2009). *AtUBC13* is involved in the plant response to DNA damage, iron deficiency and epidermal cell differentiation (Welchman et al., 2005; Wen et al., 2006; Wen et al., 2008; Li and Schmidt, 2010; Sadanandom et al., 2012). *AtUBC19* and *AtUBC20*, which belong to the *E2-C* gene family, potentially contribute to protein ubiquitination reactions and play key functional roles in the cell cycle in differentiating or differentiated cells (Criqui et al., 2002). However, *AtUBC21* (*AtPEX4*) specializes in protein for ubiquitination in peroxisome maintenance (Zolman et al., 2005). *AtUBC22* has a function in female gametophyte development and potentially in Lys11-linked ubiquitination (Wang et al., 2016). *AtUBC24* regulates the uptake, allocation as well as remobilization of inorganic phosphate (Dong et al., 1998; Lin et al., 2009), and is also the target gene of miR399s (Aung et al., 2006). *AtUBC32* is a part of the endoplasmic reticulum-associated protein degradation (ERAD) complex, which is involved in salt stress tolerance mediated by brassinosteroid (BR) (Cui et al., 2012) and in plant growth promotion in *Arabidopsis*. Moreover, the viral pathogen *Zymoseptoria tritici* induced silence of *Triticum aestivum* ubiquitin-conjugating enzyme 4 (*TaU4*) gene, resulting in postponed progression of disease symptoms, and limited reproduction as well as the growth of Septoria in wheat leaves (Millyard et al., 2016). In *Vitis vinifera*, the *UBC* family is involved in the berry ripening process and cold and heat stress responses (Gao et al., 2017). In conclusion, *UBC* genes in plants play various important roles related to stress response and plant growth.

Allotetraploid *Brassica napus* (A_n_A_n_C_n_C_n_, 2n = 38) is a globally important oilseed crop, which is derived from the hybridization between *Brassica rapa* (A_r_A_r_, 2n=20) and *Brassica oleracea* (C_o_C_o_, 2n=18) followed by chromosome doubling approximately 7500 years ago (Rana et al., 2004; Lu et al., 2011; Chalhoub et al., 2014; Sun et al., 2017; Lu et al., 2019; Wu et al., 2019). During evolution, *B. napus* and other *Brassica* species underwent multiple rounds of whole-genome duplication events (Chalhoub et al., 2014; Liu et al., 2014), and thus serve as an ideal polyploid model for studying polyploid genome evolution including gene family expansion and gene sequence and function divergence (Liu et al., 2014; Cheng et al., 2018). High-quality whole genome sequences of *B. rapa* (Zhang et al., 2018), *B. oleracea* (Belser et al., 2018) and *B. napus* (Chalhoub et al., 2014) have been published, which provide an opportunity to systematically investigate a specific gene family in *B. napus*.

So far, several studies have been performed to detect and analyze the UBC proteins on a genome-wide level in different plant species including 53 *UBC*s in sorghum (Jia et al., 2019), 57 in potato (Liu et al., 2019), 40 in longan (Jue et al., 2018), 39 in rice (E et al., 2015), 75 in maize (Jue et al., 2015), 34 in papaya (Jue et al., 2017), 59 in tomato (Sharma and Bhatt, 2017) and 74 in banana (Dong et al., 2016). However, systematic investigation of the *UBC*s in *B. napus* has been lacking. In this research, 200 *BnUBC*s were detected in *B. napus*, and their phylogenetic relationship, gene structure, conserved motif, *cis*-acting regulatory element, duplication pattern and target genes of miRNA were also systematically analyzed. The evolutionary history of the *UBC* family was explored through synteny analysis between *B. napus*, *B. rapa*, *B. oleracea*, and *Arabidopsis*. Additionally, the RNA-seq data of various tissues of *B. napus* were collected from publicly available databases for exploring the expression patterns of *BnUBC* genes. Furthermore, the genetic variations of *UBC* genes in germplasm from a global core collection of *B. napus* (Tang, 2019) were also investigated. The analysis of association mapping uncovered that some *UBC* genes were significantly associated with agronomic traits related to oil content and yield in *B. napus*. The results of this study will help us better understand the *BnUBC*s and lay the groundwork for future research on gene function and genetic breeding.

## Materials and methods

### Genome-wide identification and characterization of *BnUBC*s

Genome sequence and annotation data of *B. napus* cv. ‘Darmor-*bzh*’ (v_5.0) were obtained from the Genoscope database (http://www.genoscope.cns.fr/brassicanapus/) (Chalhoub et al., 2014). *B. rapa* ‘Chiifu’ (v3.0) datasets were obtained from the database (https://bigd.big.ac.cn/gwh/Assembly/134/show) (Zhang et al., 2018) and *B. oleracea* ‘HDEM’ (broccoli) datasets were acquired from Bolbase database (http://www.ocri-genomics.org/bolbase/index.html) (Belser et al., 2018). The ubiquitin-conjugating enzyme (UQ_con) domain (PF00179) annotation file obtained from the Pfam database (https://www.ebi.ac.uk/interpro/) was applied as queries to perform HMM search against protein sequences of annotated genes using HMMER version 3.1 (http://hmmer.org/) (Eddy, 2011) with E-value cutoff of 1e-5 (Finn et al., 2011). The amino acid sequences of *UBC*s (predicted above) were utilized as queries to carry out BLASTP searches against the full-length protein sequences of 48 *AtUBC*s from The Arabidopsis Information Resource (TAIR) database (http://www.arabidopsis.org/) and 39 *OsUBC*s from Rice Genome Annotation Project (http://rice.plantbiology.msu.edu/downloads_gad.shtml) with E-value < 1e-5. The putative *BnUBCs* with hits of both AtUBCs and OsUBCs were deployed for further confirming the existence of the UQ_con domain using the Pfam (https://www.ebi.ac.uk/interpro/), SMART (http://smart.embl-heidelberg.de/) and CDD (https://www.ncbi.nlm.nih.gov/Structure/cdd) databases. The molecular weight (MW), isoelectric point (PI), instability index, aliphatic index and grand average of hydropathicity (GRAVY) of UBC proteins, were calculated using the ProtParam tool ExPASy (https://web.expasy.org/protparam/). The subcellular location of *BnUBCs* was predicted using Plant-mPLoc (http://www.csbio.sjtu.edu.cn/bioinf/plant-multi/#) (Chou and Shen, 2010).

### Analyses of the chromosomal localization, gene structure, conserved motif and *cis*-acting regulatory element for *BnUBC*s

The chromosomal location, coding sequence (CDS) and amino acid sequences were determined based on the genome annotation of *B. napus* in the Genoscope database (http://www.genoscope.cns.fr/brassicanapus/). The chromosomal distribution of *BnUBC*s was graphically represented using the RIdeogram package of the R software (https://github.com/TickingClock1992/RIdeogram) (Hao et al., 2020). Multiple alignments of the BnUBC protein sequences were performed using CLUSTAL v2.1, with default parameters. A schematic diagram of *BnUBC*s gene structure was conducted by Gene Structure Display Server 2.0 (http://gsds.cbi.pku.edu.cn/) (Hu et al., 2015). Conserved motifs in the BnUBC proteins were investigated using the online MEME server (http://meme-suite.org/tools/meme) with the following parameters settings: the maximum number of motifs, 10; minimum width of motifs, 6; maximum width of motifs, 100 aa; and E-value < 1e-10 (Bailey et al., 2009). Moreover, the 2-kb upstream sequence of the *BnUBCs* gene sequences was extracted and submitted to PlantCARE (Lescot et al., 2002) for the detection of the *cis*-elements.

### Phylogenetic and synteny analysis of *BnUBC* proteins

To better understand the evolutionary relationships among the UBC of *B. rapa*, *B. oleracea*, *B. napus* and *A. thaliana*, a phylogenetic analysis was performed. A phylogenetic tree was constructed applying MEGA 5.2 software (Tamura et al., 2011), based on the neighbor-joining (NJ) method and 1,000 bootstrap replications. The online software Interactive Tree Of Life (iTOL, http://itol.embl.de/) (Letunic and Bork, 2016) was used to decorate this phylogenetic tree. Genes orthologous between *B. napus* and its ancestors (*B. rapa*, *B. oleracea*, and *A. thaliana*) were identified by BLASTn searches of their CDSs based on two criteria: coverage of sequence length > 80%, and identity of aligned regions > 80% (Kong et al., 2013). In addition, DupGen_finder (https://github.com/qiao-xin/DupGen_finder) (Qiao et al., 2019) was employed to identify the modes of gene duplication of *UBC* paralogous genes in *A. thaliana, B. napus, B. rapa* and *B. oleracea*. The syntenic relationship among these paralogs was presented using the fmsb package of the R software.

### Ka/Ks calculation

The KaKs calculator (Wang et al., 2010) was applied to estimate the divergence between pairwise nonsynonymous substitution rates (Ka) and synonymous substitution rates (Ks). The evolutionary constraint (Ka/Ks) of *UBC* orthologous genes between *B. napus* and the other three species (*A. thaliana*, *B. rapa* and *B. oleracea*) was calculated according to their CDSs. In addition, to minimize errors, only the gene pairs with Ks < 1 were remained for further analysis (Guo et al., 2017; Wang T, et al., 2020).

### Prediction of miRNAs targeting *BnUBCs*

The psRNATarget Server (Dai et al., 2018) was used to predict the miRNAs potentially targeting *BnUBC*s. The gene sequences of *BnUBCs* were submitted as candidates to search against the available sequences of reference *B. napus* miRNA with default arguments. A network of interactions between the predicted miRNAs and their target *UBC* genes in *B. napus* was visualized using the Cytoscape software (Shannon et al., 2003).

### Expression profiles and gene ontology enrichment analysis of *BnUBCs*

The publicly available RNA-seq datasets of five different *B. napus* tissues (callus, root, leaf, bud and silique) were collected from the NCBI SRA database (accession no. SRP136038) (Yao et al., 2020). The expression levels of *BnUBC*s were quantified based on their fragments per kilobase of exon per million reads mapped (FPKM) values using Cufflinks with default parameters (Trapnell et al., 2012). Furthermore, the expression levels of *BnUBC* genes were used to construct clustered heatmaps with TBtools (Chen et al., 2020). Then, GO enrichment analysis of *BnUBC* genes was performed using the clusterProfiler package of the R software.

### Association mapping of *UBC* genes in a natural population of *B. napus*

The agronomic traits of 324 worldwide accessions comprising a natural population of *B. napus* were applied to detect the natural sequence variations of *BnUBCs* (Tang, 2019; Xie et al., 2022). Single nucleotide polymorphisms (SNPs) in *BnUBCs* were annotated using the SnpEff software (Cingolani et al., 2012). Moreover, agricultural traits involving the primary flowering time (PFT), full flowering time (FFT1), final flowering time (FFT2), plant height (PH), thousand seed weight (TSW), main inflorescence silique density (MISD), main inflorescence silique number (MISN), oil content (OC), protein content (PC) and main inflorescence length (MIL) were used to conduct associated mapping (Tang, 2019). The EMMAX software was applied to perform family-based association mapping analysis with a mixed linear model (Kang et al., 2010). Then, the visualization of haplotype blocks and linkage disequilibrium was conducted using the LDBlockShow software (Dong et al., 2021b). The interaction networks of *B. napus* proteins were obtained from the STRING database (Damian et al., 2020).

## Results

### Genome-wide identification and characterization of *BnUBC*s

To identify the members in the *UBC* family, the annotation file of UBC domain obtained from the Pfam database (http://pfam.xfam.org/) was applied as a query for searching against the protein dataset of *B. napus*. The peptides of assumed *BnUBC*s showing hits of both *AtUBCs* and *OsUBCs* were further employed to confirm the presence of the UBC domains by searching in the Pfam, SMART and CDD databases. In total, 200 *BnUBC*s were identified in *B. napus* (**Supplementary Table 1**). The full-length transcript sequences of *BnUBCs* ranged from 168 bp (*BnaA08g09170D*) to 5,656 bp (*BnaC07g05960D*) with the deduced amino acid sequences varying from 55 aa (*BnaA08g09170D*) to 1,649 aa (*BnaA07g03330D*) (**Supplementary Table 1**). The predicted MW of the 200 deduced BnUBC proteins ranged from 6.10 kDa (*BnaA08g09170D*) to 184.41 kDa (*BnaA07g03330D*), and their GRAVY and pIs ranged from -0.904 (*BnaC04g10080D*) to 0.112 (*BnaA08g13930D*) and from 4.19 (*BnaA04g11090D*) to 9.57 (*BnaA09g09660D*), respectively (**Supplementary Table 1**). As previously reported in other plant species (Die et al., 2018; Zhou et al., 2018), a wide range of pIs suggested that BnUBCs function in different subcellular environments. Furthermore, the instability index of 38 of the 200 BnUBC proteins was less than 39, and these proteins were classified as stable. In addition, the prediction of their subcellular localization indicated that 172 BnUBCs were located in the nucleus, 27 in the cytoplasm and the other one in the cell membrane. In addition, to explore the evolutionary relationships among members of this family across Brassicaceae crops, 93 *BraUBCs* and 95 *BolUBCs* were identified in the reference genomes of *B. rapa* and *B. oleracea* respectively, following the same identification pipeline (**Supplementary Table 2**). Details of these *BraUBCs* and *BolUBCs* were listed in **Supplementary Table 2**.

### Chromosomal distribution analysis of *BnUBC*s

**Figure 1:**
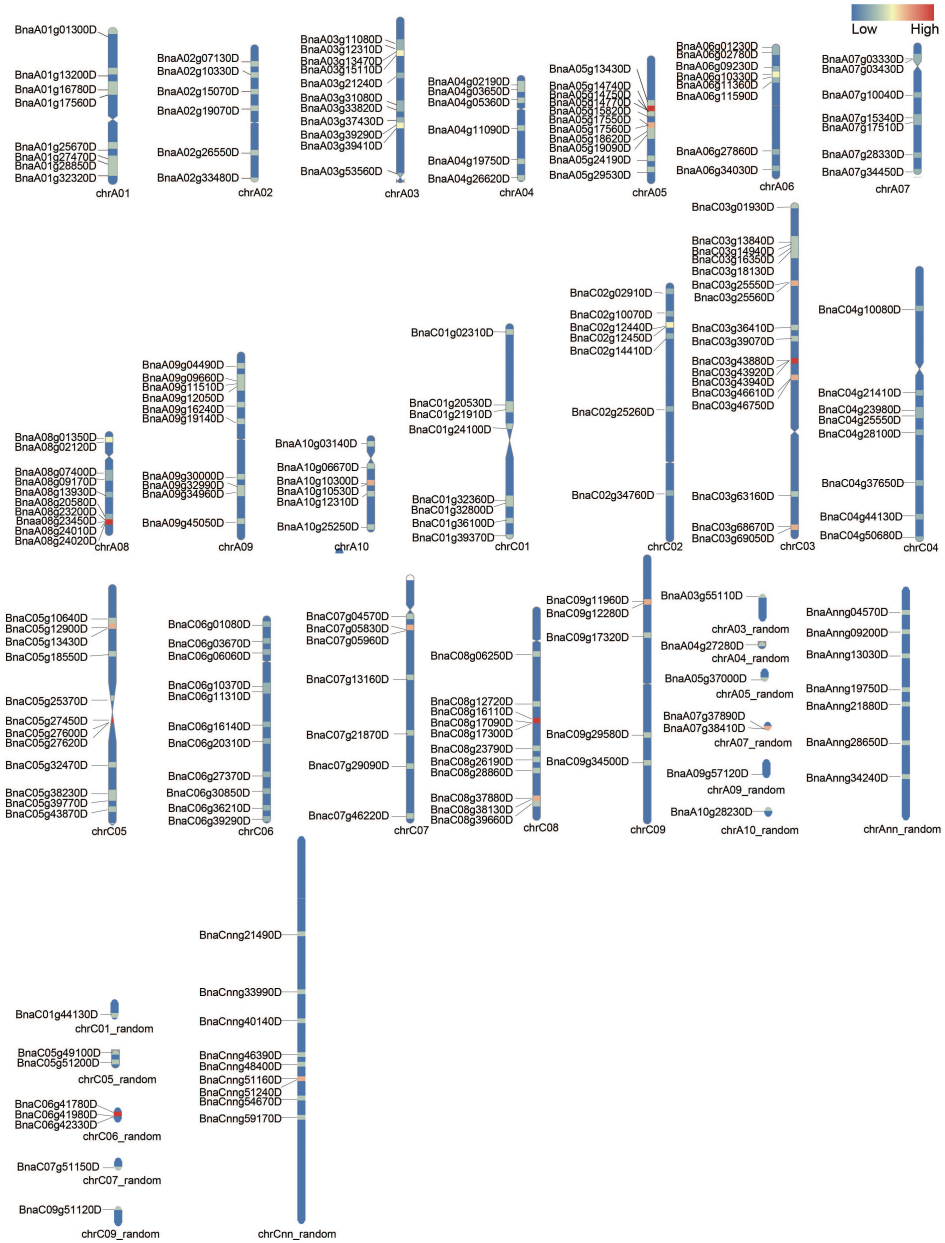
Chromosomal distribution of *BnUBC*s. The *BnUBCs*’ genome locations are plotted based on the location of genes, length of chromosomes, and positions of centromeres. The chromosome name is marked at the bottom of each bar. Heatmap represents the gene density (number of genes per Mb) of each chromosome.

The chromosomal positions of detected *BnUBC*s were drafted to corresponding chromosomes with the RIdeogram package of R software. The 200 *BnUBC*s were unevenly distributed across all 19 chromosomes with 97 and 103 genes located in the A_n_ and C_n_ subgenomes, respectively (**Figure 1**). The A_n_ subgenome carried, on average 9.7 *BnUBCs* on 10 chromosomes; A10 had the lowest number of *BnUBC*s (6), while A03 and A05 harbored the highest of *BnUBC*s (11 each). The average number of *BnUBCs* in the C_n_ subgenome was 11.4, with the lowest number (5) on C09 and the highest number on C03. Hence, no biased tendency was observed between the two subgenomes. Furthermore, on each chromosome, *BnUBCs* were unevenly distributed, especially, since the genes on A06 and A08 were located at the ends.

### Phylogenetic relationships analysis of *BnUBC*s

**Figure 2:**
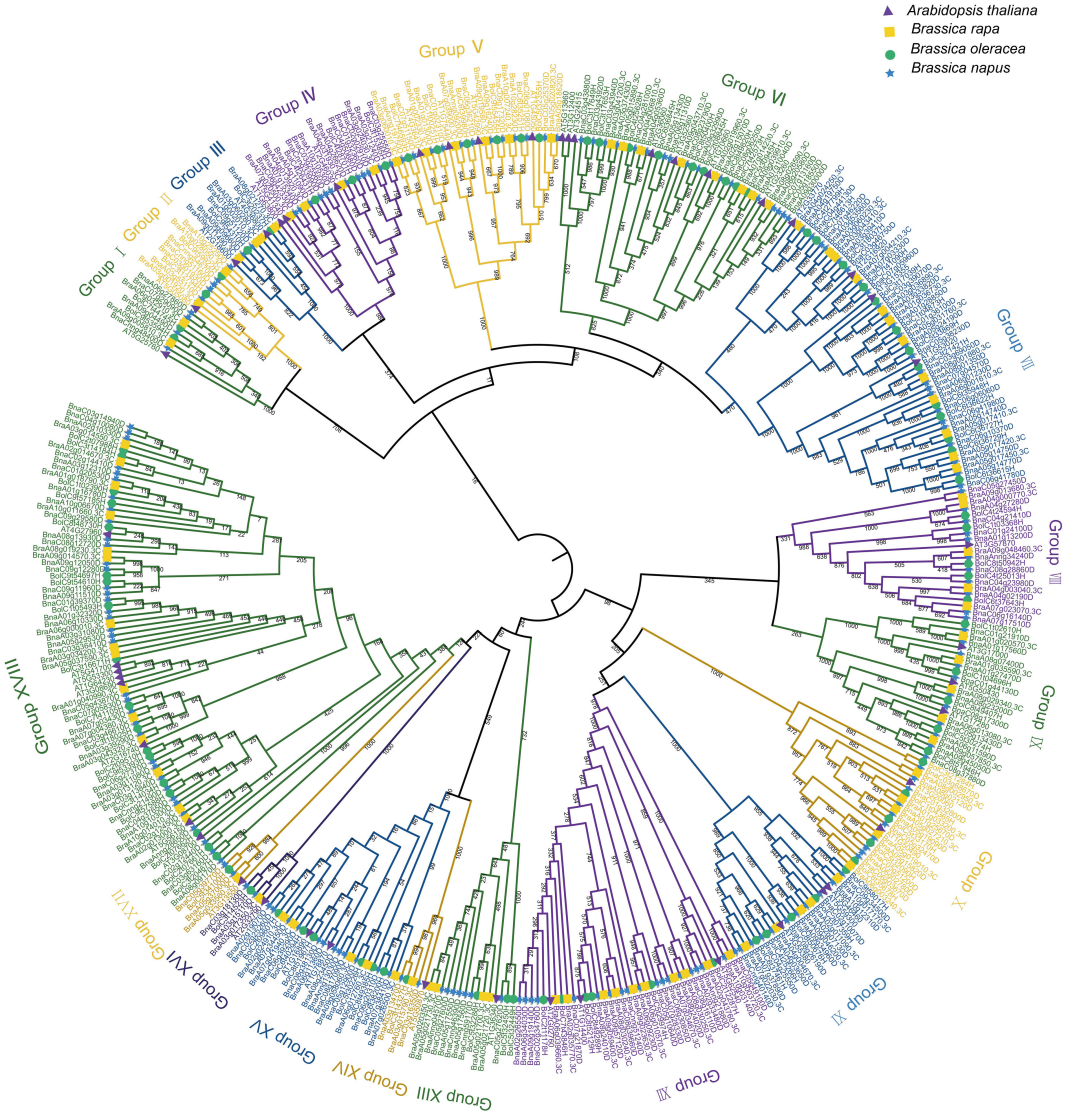
The phylogenetic relationship of *UBC* genes among *A. thaliana*, *B. rapa*, *B. oleracea* and *B. napus*. The construction of a phylogenetic tree using the *UBC* gene family among *A. thaliana*, *B. rapa*, *B. oleracea*, and *B. napus*. MEGA7.0 generated the phylogenetic tree topology using the neighbor-joining method with 1,000 bootstrap replicates. The members belonged to the same subgroup according to the node and branch they located and the characteristic of the tree. The different subgrouops were depicted in various colors.

To explore the evolutionary relationships among the *UBC* genes in *A. thaliana*, *B. rapa*, *B. oleracea*, and *B. napus*, a tree of phylogenetic was constructed based on the multiple sequence alignments of 48 *AtUBC*s, 93 *BraUBC*s, 95 *BolUBC*s, and 200 *BnUBC*s. All 200 *BnUBC*s could be categorized into 18 subfamilies and the number of groups was consistent with a previous report (Jia et al., 2019) (**Figure 2**). Among them, Group XVIII possessed the highest number (37) of the *BnUBC*s*s*. Furthermore, group III (two *BnUBC*s) and group VIII (ten *BnUBC*s) members were involved in RUB and SUMO conjugation pathways, respectively, and other three subgroups (XVI, XV, VI) contained the members lacking the Cys active-site (UEVs).

### Gene structure, conserved motifs as well as *cis*-acting regulatory elements analysis of *BnUBCs*

**Figure 3:**
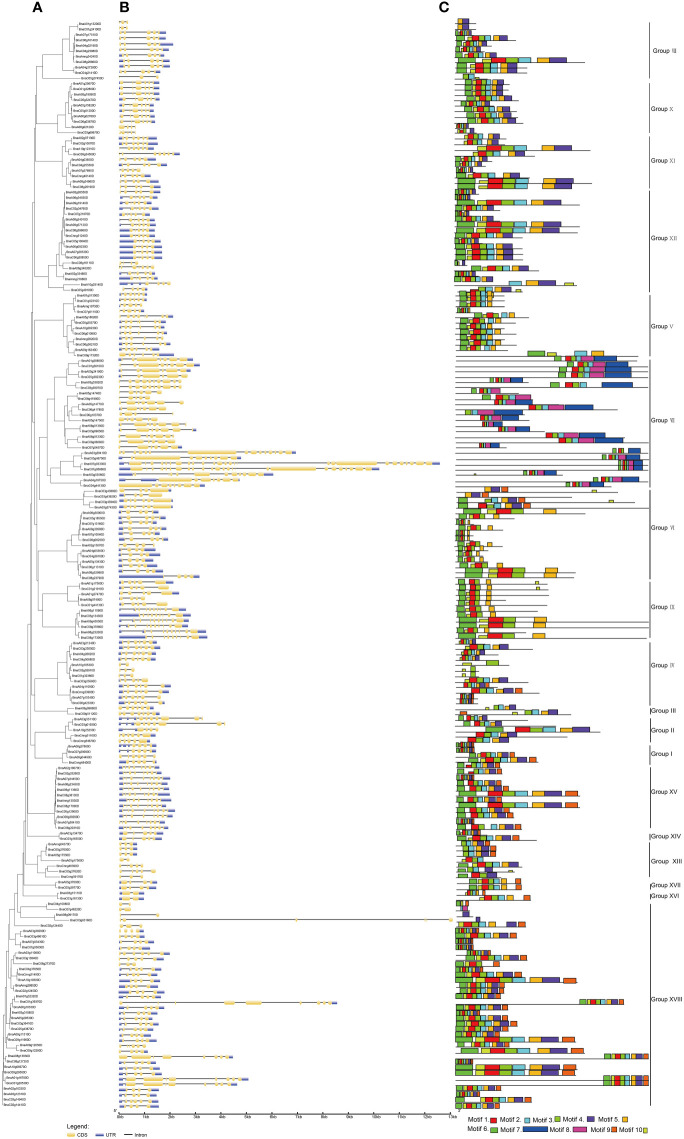
Gene structure and motif analysis of *B. napus UBC* genes according to their phylogenetic relationships. **(A)** Phylogenetic analysis. **(B)** Gene structures of *BnUBC*s. Yellow and blue-filled boxes represented CDS and UTR, respectively. Black lines indicated introns. **(C)** Conserved motifs in BnUBC proteins were detected by MEME analysis.

Concerning the evolution of multigene families, the variety of gene structures provided an important resource (Ischoff et al., 2002; Yunpeng et al., 2016; Pellicer et al., 2018). For exploring the multiplicity of the gene structure of *BnUBCs* among the different groups, the intron-exon structure of *BnUBC*s was compared based on the phylogenetic relationships of these genes (**Figure 3A**, **Supplementary Table 3**). The results indicated that the gene architecture of *BnUBC*s was relatively complex (**Figure 3B**). Only five genes (*BnaA10g10530D*, *BnaA05g17560D*, *BnaC04g10080D*, *BnaC07g46220D* and *BnaA08g09170D*) contained a single intron, while the others harbored multiple introns. *BnaA07g03330D* contained the most introns and exons. Besides, 33 of 200 *BnUBC*s lacked untranslated regions (UTR) at both ends or one end because of inaccurate annotation. The number of introns among the *BnUBCs* ranged from 1 to 26, and the length of introns ranged from 14 to 6,632 bp. Moreover, exon numbers in*BnUBC*s also varied widely across distinct subfamilies, ranging from 2 to 26. However, the majority of the members within the same subfamily tended to show similar intron-exon distribution patterns. For example, members of Group X generally possessed five exons, whereas those of Group XV, harbored eight exons. Therefore, the organization of introns and exons presented valuable evidence for the phylogenetic relationship among members of this gene family.

Furthermore, a total of 10 conserved motifs were recognized in 200 *BnUBC* genes (**Figure 3C**). Conserved motifs ranged in length from 11 to 74 aa, and among 10 motifs motif 3 as well as motif 1 were the most abundant. Moreover, the motif 1-4 were significantly matched to the UQ_con domain (Pfam: PF00179). Furthermore, a motif sequence search in the CDD database revealed that motifs 5 and 6 belonged to UBCc family. In addition, motifs 7-8 and motif 10 were matched to UBQ-conjugating_enzyme/RWD based on the search in the InterPro database. Motif composition varied across distinct subfamilies, whereas the conserved character of motif distributions in the same subcategory was similar, which emphasized their phylogenetic relationship. For example, all Group IX members possessed motif 1, motif 4-6 and motif 10, whereas in Group X, members generally contained motifs 1-6 and motif 10. In addition, three structural properties were detected and refined in BnUBC proteins (**Supplementary Figure 1**).

The *cis*-acting elements in the promoter region of a gene can modulate the initiation and efficiency of gene expression through the binding of specific transcription factors, associated with plant development, growth and stress response (Ibraheem et al., 2010). To better understand the potential functions and transcriptional regulation of *BnUBC*s, we isolated the sequences 2-kb upstream of the *BnUBC* CDSs from the *B. napus* genome sequence to search for *cis*-elements (**Supplementary Table 4**) (Chalhoub et al., 2014). A total of 115 functional *cis*-elements were detected in the promoter regions of *BnUBCs* (**Supplementary Figure 2**, **Supplementary Table 5**). Among these *cis*-elements, many were light-responsive and hormone-related related elements. Besides, majority of *BnUBC*s (197/200, 98.50%) contained both CAAT-box and TATA-box elements, which usually existed in eukaryon. Moreover, the MYB element, which participates in the regulation of phenylpropanoid secondary metabolism in plants was identified in 192 out of 200 *BnUBCs*. The MYC element was identified in 183 of the 200, (91.50%) *BnUBC*s, which were involved in the regulation of plant growth and development as well as resistance to environmental stress and secondary metabolite synthesis. Furthermore, many *BnUBCs* contained light-responsive elements such as Box 4 element (part of a conserved DNA module involved in the light response), G-box element (*cis*-acting regulatory element involved in the light response), GT1-motif (light responsive element), and TCT-motif (part of a light responsive element) (**Supplementary Figure 2**, **Supplementary Table 5**). Additionally, the stress-related elements, including the LTR element and TC-rich repeats were also detected in *BnUBCs*, suggesting that the *BnUBC*s played an important role in plant development and growth as well as in response to biotic and abiotic stresses.

### Synteny and gene duplication analysis of *UBCs* in *B. napus, A. thaliana, B. rapa* and *B. oleracea*

**Figure 4:**
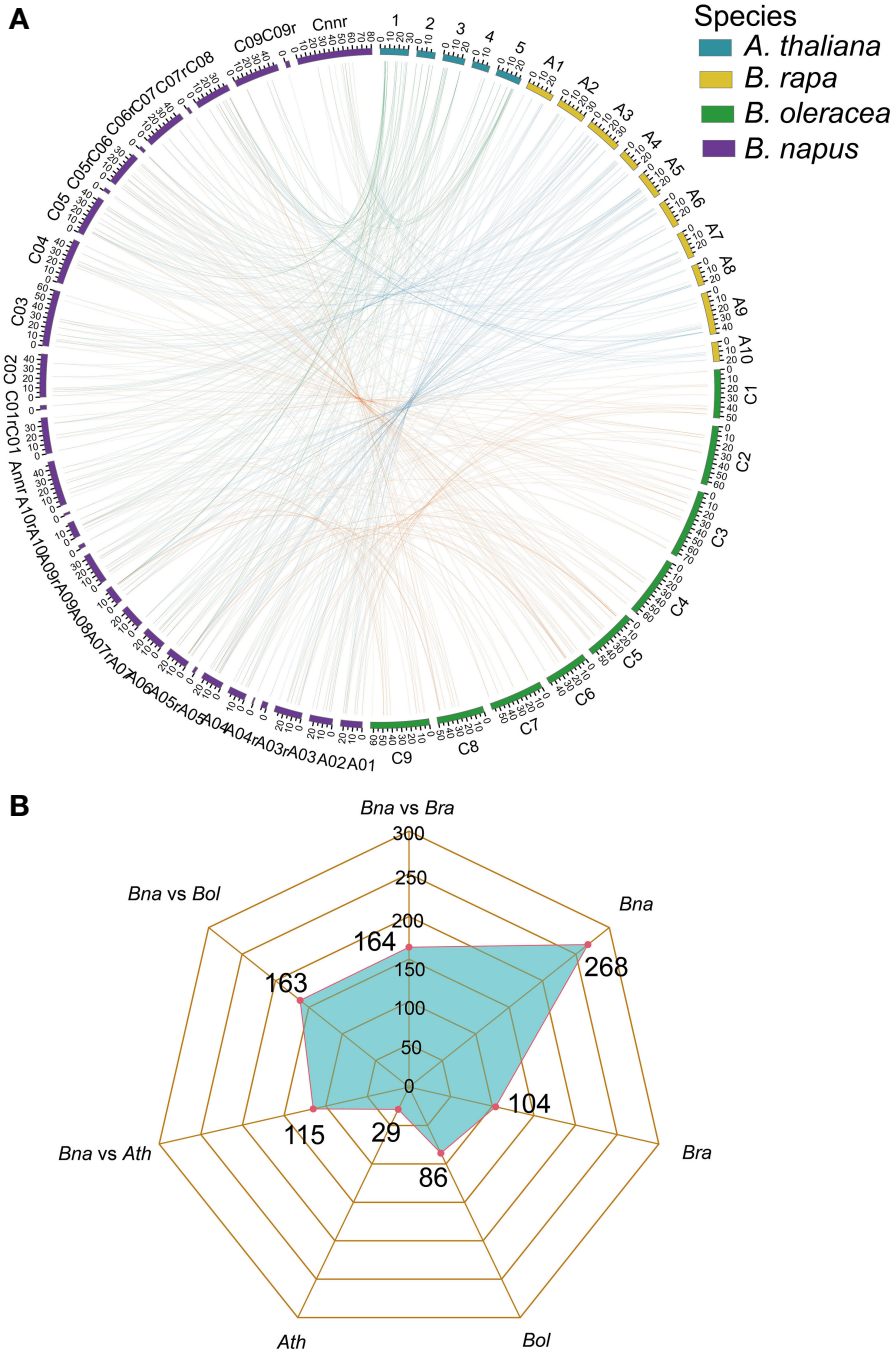
Genome-wide synteny analysis of *UBC* genes among *A. thaliana*, *B. napus*, *B. rapa*, and *B. oleracea* **(A)** Collinearity among the *UBC* genes in *A. thaliana*, *B. rapa*, *B. oleracea*, and *B. napus*. The chromosomes of *A. thaliana*, *B. rapa*, *B. oleracea* and *B. napus* were indicated with different colors corresponding to the color legend at the top right. Green lines represented the collinearity between *A. thaliana* and *B. napus*. The blue lines represented the collinearity between *B. rapa* and *B. napus*. The orange lines represented the collinearity between *B. oleracea* and *B. napus*. The visualization of the collinear correlations was performed by CIRCOS software. **(B)** Radar charts showed the number of orthologous and paralogous gene pairs of *UBC*s across four plant species.

Gene family expansion occurred mainly *via* the duplication of genes through whole-genome duplication (WGD), tandem duplication as well as segmental duplication events (Bodt et al., 2005; Mun et al., 2009). Moreover, the duplication of ubiquitin members through WGD events plays important role in plants (Hua et al., 2018). To further explore the evolutionary history of *BnUBC* gene family expansion we performed a syntenic comparison of genome sequences between *A. thaliana*, *B. napus*, *B. rapa* and *B. oleracea*. *A. thaliana* is the ancestor of *Brassica* species, whose structural genes have been identified and functionally annotated, therefore it is served as a prominent model system for the investigation of the evolutionary history of *Brassica*. homologous genes from inter-species comparison and paralogous genes from intra-species comparison were identified to trace the duplicated gene pairs of *BnUBC*s. We identified 268, 104, 86 and 29 paralogous *UBC* gene pairs of within *B. napus*, *B. rapa*, *B. oleracea* and *A. thaliana*, respectively (**Figure 4B**). Out of 286 paralogous *UBC* gene pairs in *B. napus*, 50 and 44 pairs were detected in the A_n_ subgenome, and C_n_ subgenome, respectively, while the remaining 174 pairs were identified across the A_n_ and C_n_ subgenomes. Moreover, we classified these paralogous gene pairs into five types: dispersed, proximal, tandem, WGD and transposed (**Table 1**, **Supplementary Table 6**). In *B. napus*, 195 genes, accounting for 97.5% of all *BnUBC*s, were derived from gene duplication, of which WGD (64.5%) and transposed (17.4%) gene duplication events were primarily responsible for gene family expansion (**Table 1**, **Supplementary Table 6**).

**Table 1:** The identification of duplicated type for *UBC* genes in *B. napus* and other three Brassicaceae species.

Species	WGD	Tandem	Proximal	Transposed	Dispersed	Total
*B. napus*	221	1	1	45	0	268
*B. rapa*	82	1	2	19	0	104
*B. oleracea*	66	1	2	17	0	86
*A. thaliana*	13	1	0	14	1	29

**Figure 5:**
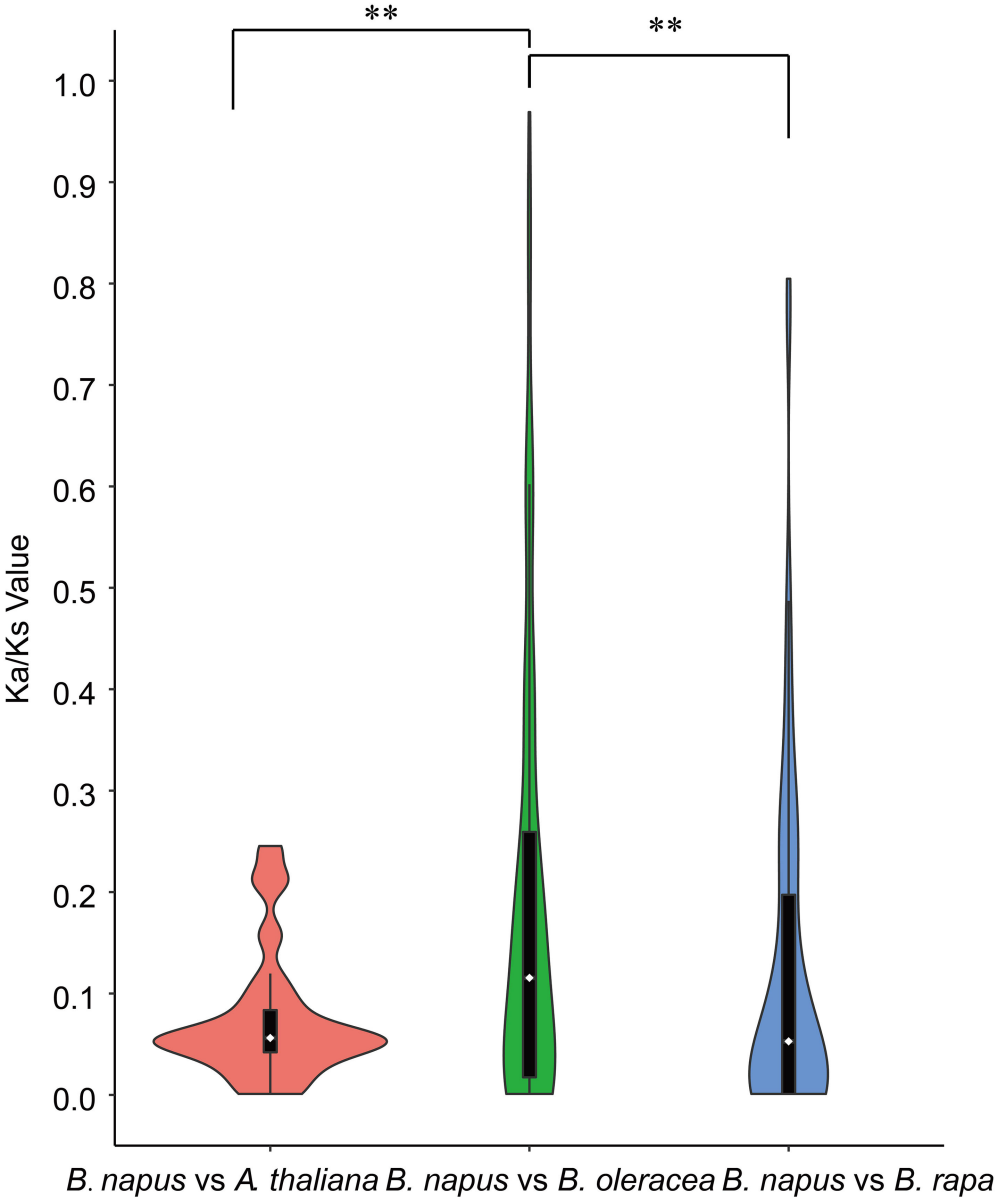
Violinplot of the pairwise Ka/Ks ratios of syntenic orthologous gene pairs. Only orthologous gene pairs with Ks <1 were considered. Wilcoxon Rank-Sum Tests were performed between different types of orthologous gene groups (**P < 0.01). Dotted lines within the violin plots represented the first, second, and third quartiles.

Furthermore, synteny comparison between *B. napus* and the other three species revealed that 180 *BnUBCs* were collinear to the orthologous genomic regions in one or more of the other three species, 164 orthologous gene pairs were identified between *B*. *napus* and *B*. *rapa*, 163 between *B*. *napus* and *B*. *oleracea* as well as 115 between *B*. *napus* and *A*. *thaliana* (**Figures 4A, B**, **Supplementary Table 7**). It was obvious that majority of the *BnUBC*s were inherited from their progenitors. In addition, *Brassica* species experienced an extra whole-genome triplication (WGT) event after divergence from *A. thaliana* (Feng et al., 2014). Therefore, a single *A. thaliana* gene was inherited as three copies in *B. oleracea* as well as *B. rapa* and as six copies in *B. napus*, if no gene loss occurred after the WGT event. We found that only 25.9% of the *AtUBC*s corresponded to six copies in *B. napus*, suggesting that a substantial gene loss occurred during the process of polyploidization. To estimate the selection pressure on *BnUBC*s, the non-synonymous to synonymous substitution ratios (Ka/Ks) of *BnUBC* orthologous genes to *BraUBC*s, *BolUBC*s, and *AtUBC*s were calculated. The Ka/Ks less than one represents purifying selection, whereas Ka*/*Ks equal one indicates neutral evolution and Ka*/*Ks more than one represents positive selection (Nekrutenko, 2002). Thus, the Ka/Ks ratio can predict the pressure of selection on each duplicated pair throughout evolution as well as their divergence time. The Ka/Ks ratios of all the *BnUBCs* paralogous gene pairs, except two duplicated gene pairs (*BnaA07g03430D*/*BnaC07g05830D*, *BnaC02g02910D*/*BnaC03g25550D*) were less than one, suggesting that these genes were subjected to strong purifying selection (**Supplementary Table 8**). Moreover, the duplicated gene pair *BnaA07g03430D*/BnaC07g05830D had Ka/Ks ratio considerably larger than 1, suggesting these genes were subjected to strong evolutionary pressure under positive selection. Furthermore, the Ka and Ks values of the identified orthologous gene pairs between *A. thaliana* and *B. napus* were calculated to estimate the selection pressure on orthologous gene pairs and the divergence time of the two species. The Ka/Ks ratios of all the orthologous gene pairs ranged from 0.001 to 0.25 with an average of 0.074, which indicated these genes were under purifying selection (**Supplementary Table 9**). The orthologous gene pairs among *A. thaliana* and *B. napus* diverged around 16 million years ago (MYA) based on the mutational rate estimate, R = 1.5 × 10^-8^ synonymous substitutions per site per year (Koch et al., 1999; Koch et al., 2001). This result was consistent with the previously estimated divergence time (14–24 MYA) between the *A. thaliana* and *B. napus* lineages (Koch et al., 2000; Cheung et al., 2009). In addition, the Ks values of the orthologous genes between *B. napus* and its progenitors were also calculated (**Supplementary Table 9**). The Ka/Ks ratio of the orthologous gene pairs between *B. napus* and *B. oleracea* was significantly higher than that between *B. napus* and *B. rapa*, showing that genes in the C_n_ subgenome underwent relatively weaker selection pressure in *B. napus* during the process of evolution (**Figure 5**). In addition, the Ka/Ks values of the orthologous gene pairs between *B. napus* and *Arabidopsis* were significantly lower than those of the orthologous gene pairs identified between *B. napus* and *B. oleracea* and between *B. napus* and *B. rapa*, indicating that orthologous gene pairs between *B. napus* and *A. thaliana* underwent intense purifying selection. Moreover, the sequences of their proteins might maintain more consistent characteristics during evolution (**Figure 5**).

### Comprehensive analysis of miRNA-targeting *BnUBC*s

**Figure 6:**
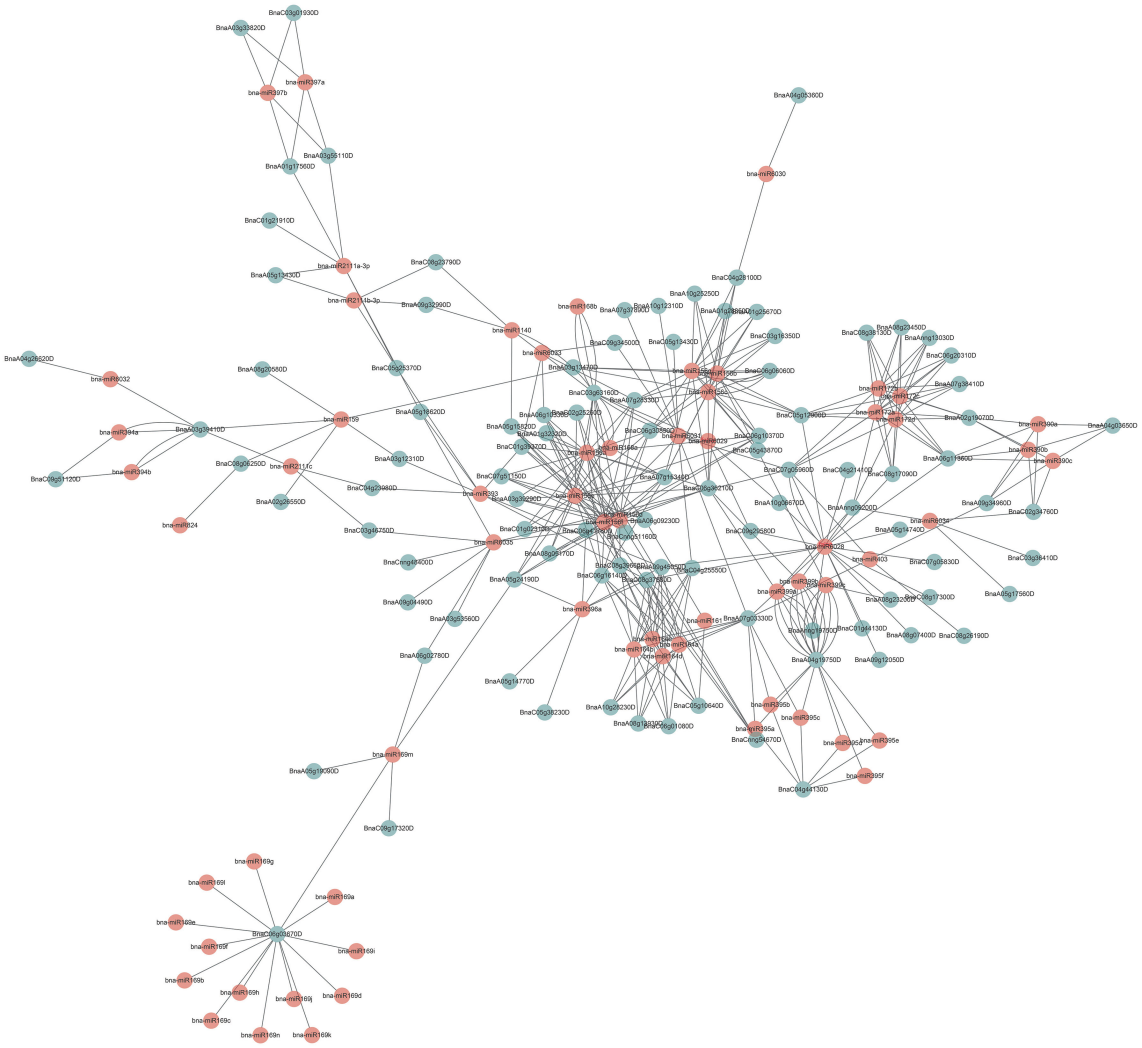
Schematic representation of the interaction network between the miRNAs and their putative *BnUBC* targets.

Many miRNAs were involved in regulating the function of their targeted genes in plants (Jones-Rhoades and Bartel, 2004; Yu et al., 2017; Song et al., 2019). To explore the miRNAs regulating *BnUBCs*, 106 *BnUBC* targeting genes of 71 putative miRNAs were identified, and their relationship network was constructed by Cytoscape software (**Figure 6**, **Supplementary Table 10**). It was found that *BnaC06g03670D* was targeted by the *B. napus* miRNA169 family based on an interaction network. A recent study showedthat miR169 regulated the function of the anaphase-promoting complex/cyclosome (APC/C), an essential ubiquitin-protein ligase, by targeting DUO POLLEN1 (DUO1). DUO1 upregulated the expression level of APC/C, which stimulates the production of miR159 (Zheng et al., 2011). Moreover, members of the miRNA156 family were found to regulate most of the *BnUBCs*. In a recent study, overexpression of miR156 inhibited gibberellic acid (GA)-induced and ubiquitination-mediated degradation of DELLA (Jerome Jeyakumar et al., 2020). In addition, the remaining *BnUBCs* were presumably targeted by members of the other 26 miRNA families (**Figure 6**).

### The expression patterns of *BnUBC*s in various tissues and gene ontology analysis of *BnUBC* proteins

Several studies reported that members of the *UBC* family mediated crosstalk among diverse signaling pathways for various abiotic stress responses (Gao et al., 2017; Jue et al., 2018; Jia et al., 2019). Besides, *UBCs* were predicted to play an important role in protein and ion binding (Liu et al., 2019). To predict the putative functions of *BnUBCs*, GO enrichment analysis was performed. The GO terms were divided into three categories: biological process (BP), molecular function (MF), and cellular component (CC) (**Supplementary Table 11**). The majority of enriched GO terms, such as postreplication repair, belonged to the BP category (**Supplementary Figure 3**). The remaining GO terms were broadly associated with vegetative growth (negative regulation of flower development, root epidermal cell differentiation), response to stress (response to iron ion, cellular response to water deprivation and response to gibberellin), cell growth and protein process. The CC category included UBC13−MMS2 complex and the perinuclear region of the cytoplasm. Moreover, various MF were detected in this analysis, which included acid-amino acid ligase activity, SUMO transferase activity, ubiquitin protein ligase binding and endopeptidase activity. The results of GO enrichment analysis suggested that *BnUBCs* play crucial roles in plant development, growth, cell protein quality regulation and response to stress, consistent with the findings of previous studies (Dreher and Callis, 2007; Sadanandom et al., 2012; Jue et al., 2018).

**Figure 7:**
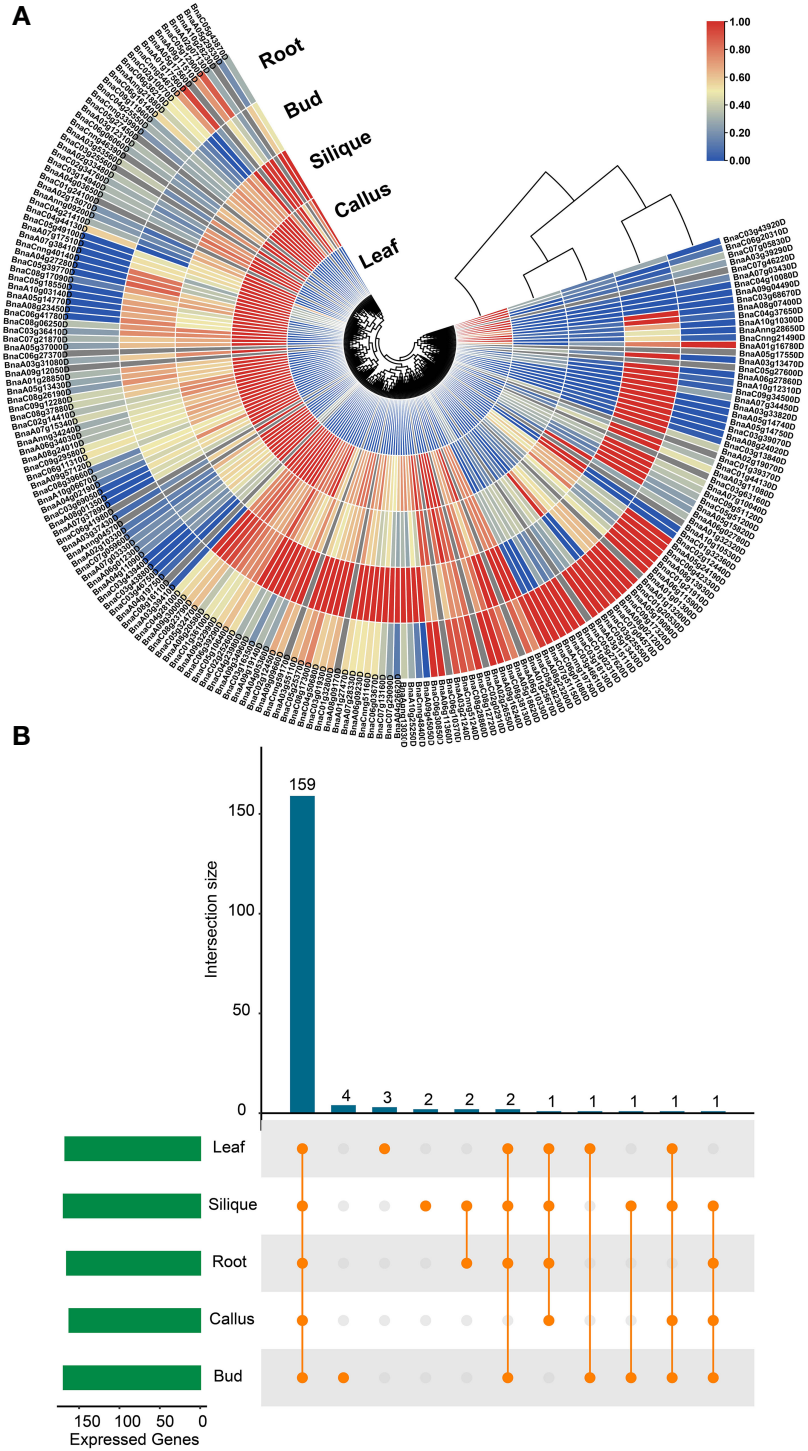
Expression profiles of *BnUBCs* across various tissues. **(A)** Expression data were represented by values of FPKM, and different colors showed different expression levels. **(B)** Number of *BnUBC*s expressed across various tissues.

Moreover, the expression profiles of *BnUBC* genes were examined across five major tissues (callus, leaf, root, bud and silique) based on the previously published RNA-seq datasets of *B. napus* (Yao et al., 2020). The expression levels of *BnUBC*s were estimated with FPKM and displayed as a heatmap (**Figure 7A**). A total of 38 *BnUBCs* showed relatively weak expression (FPKM < 1) and 24 *BnUBC*s were silience in any of the five tissues. The remaining *BnUBCs* showed high expression levels (FPKM ≥ 1), the majority of which expressed at a specific organ or differentially expressed in various tissues (**Figure 7B**). *BnaC06g16140D* (ortholog of *AT3G57870*) presented the highest expression in all tissues, suggesting its critical roles in plant growth. Genes showing low expression levels (FPKM <1) across all five tissues could be regarded as pseudogenes (**Figure 7**, **Supplementary Table 12**). Notably, in our study, most of *BnUBC*s exhibited relatively lower expression levels in the leaf than in other tissues (**Supplementary Table 12**).

Furthermore, the expression divergence between the duplicated genes in *B. napus* investigated. For example, *BnaA06g10330D* was silence across all tissues, whereas its paralog *BnaA03g31080D* presented a high expression level in various tissues. Similarly, *BnaA04g02190D* expressed in all tissues, however, its paralogs *BnaA01g13200D* and *BnaC01g24100D* didn’t express in any tissue. All these results could provide a clue for the functional divergence between duplicated gene pairs in *BnUBCs*.

### Genetic effects of *BnUBC*s on agronomic traits

SNPs in a natural population with 324 *B. napu*s accessions collected from worldwide countries were identified (Tang, 2019) (**Supplementary Table 13**) for investigating the genetic variants of *BnUBC*s in germplasm. Each *BnUBC* gene contained 34 SNPs on average, which was close to the 36 SNPs in each gene across the entire genome. Taking genome size into account, the number of SNP in each kilobase (kb) among the *BnUBCs* was calculated (17 SNPs/kb), whereas 13 SNPs/kb for the genes across entire genome. *BnUBC*s in the A_n_ subgenome contained slightly higher SNP density (20 SNPs/kb) than that in the C_n_ subgenome (14 SNPs/kb). Moreover, variations in SNP number between some paralogous gene pairs of *BnUBCs* were observed. For example, *BnaA03g11080D* possessed 65 SNPs, however, its paralog *BnaC02g12440D* contained no SNP. Moreover, the paralogs *BnaA07g03330D*/*BnaC03g46750D*, contained 235 and 33 SNPs, respectively. Based on the paired t-test, no significant difference was detected in SNP density between the *BnUBC* paralogs. Additionally, SNP annotation presented that 1,671 SNPs existed in the exon regions of *BnUBCs* and 600 SNPs lead to missense mutations.

**Figure 8:**
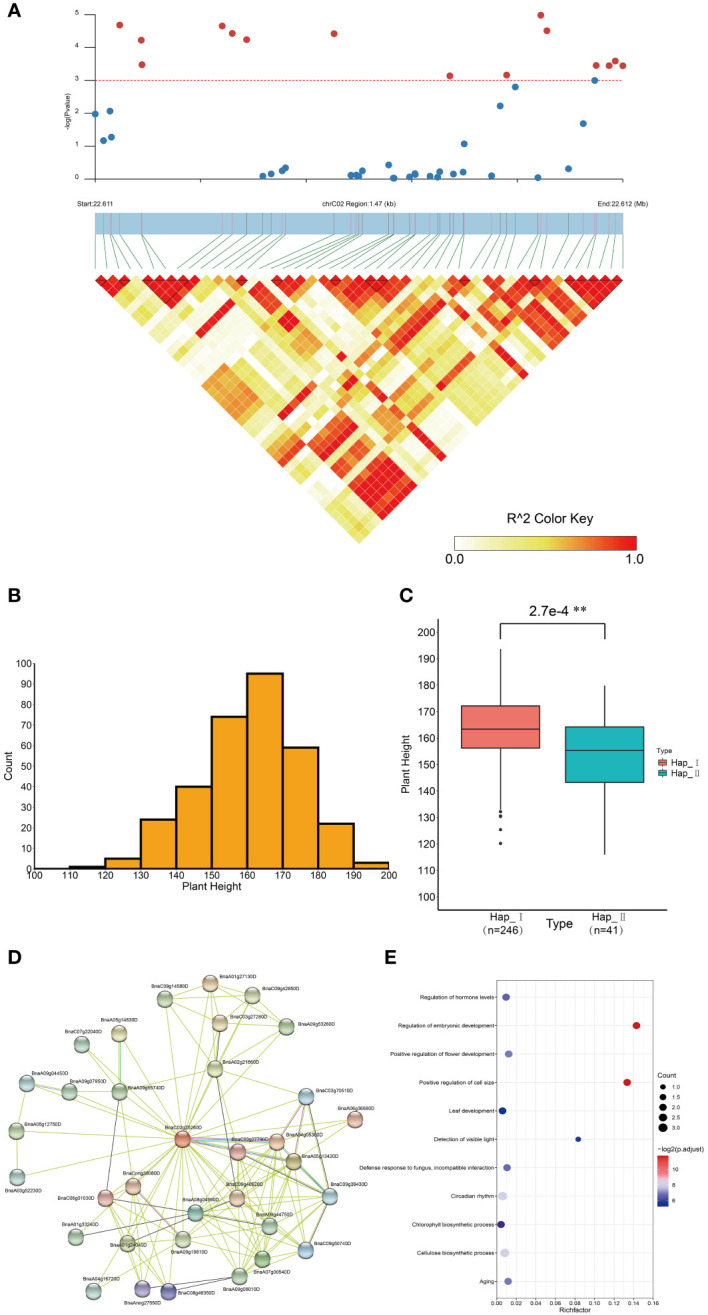
Association mapping analysis of *BnaC02g25260D* in *B. napus* germplasm with 324 core collections. **(A)** Significant association of *BnaC02g25260D* with tplant height. **(B)** Distribution of the plant height of 324 accessions. **(C)** Box plot showed the comparison of plant height between two haplotypes divided based on the most significantly associated SNP in *BnaC04g00810D*. **(D)** Protein-protein interaction network of BnaC02g25260D. **(E)** GO enrichment analysis of BnaC02g25260D interacting proteins. **p < 0.01.

Ubiquitination is a well-characterized post-translational that regulates plant growth and developmental processes contributing to diverse phenotypes, and affect agronomic traits (Dreher and Callis, 2007; Vierstra, 2009; Sadanandom et al., 2012; Linden and Callis, 2020; Yu and Hua, 2022). Association mapping analysis was performed to explore the impact of *BnUBC*s on agronomic phenotype. In total, 96 SNPs in 33 *BnUBC* genes (**Supplementary Table 13**) were significantly associated with the investigated agronomic traits (*p* < 0.001). Moreover, *BnaC02g25260D* was significantly associated with plant height (**Figure 8A, B**). Furthermore, the two genotypes of accessions were divided according to the most significantly associated SNP of *BnaC02g25260D*, and the plant height between the two genotypes was statistically significant according to the t-test (*p* < 2.7 e–4) (**Figure 8C**). The protein interaction network of *BnaC02g25260D* was obtained from STRING (Damian et al., 2020) (**Figure 8D**) and the interacted genes were enriched in the regulation of embryonic development (GO:0045995), positive regulation of cell size (GO:0045793), cellulose biosynthetic process (GO:0030244), circadian rhythm (GO:0007623), positive regulation of flower development (GO:0009911), detection of visible light (GO:0009584), leaf development (GO:0048366), regulation of hormone levels (GO:0010817) and chlorophyll biosynthetic process (GO:0015995) (**Figure 8E**). Overall, the above categories were related to plant development and growth, the regulation of hormones and oil content, which affected the plant height and the cell size in seed formation (Wang X, et al., 2020; Dong et al., 2021a). Furthermore, SNPs distributed in *BnaAnng34240D* were significantly associated with flowering time and silique density (**Supplementary Figure 4A-D, G-H**). The protein interaction networks of *BnaAnng34240D* were also obtained from STRING (**Supplementary Figure 5A**), and its interacted proteins enriched in the protein modification process, regulation of transcription and so on (**Supplementary Figure 5B**). Besides, *BnaA07g28330D* was significantly associated with TSW, which affect the crop yield (**Supplementary Figure 4E, F**). Furthermore, we found that *BnaA07g28330D* exhibited a higher expression level in the silique of high oil content accession than in that in the silique of low oil content accession (https://bnaomics.ocri-genomics.net/tools/exp-view/index.php) (Tang, 2019).

## Discussion

Ubiquitin-conjugating enzymes transfer ubiquitin from ubiquitin-activating enzymes to ubiquitin ligases, which is a key step in protein ubiquitination (Bae and Kim, 2013). In plants, UBC proteins are involved in multiple crucial processes, including growth, development and abiotic stress response (Welchman et al., 2005; Dreher and Callis, 2007; Sadanandom et al., 2012). However, only a few members of the UBC family have been characterized in plants such as *Arabidopsis* (Kraft et al., 2005), rice (E et al., 2015), maize (Jue et al., 2015) and tomato (Sharma and Bhatt, 2017) on the genome-wide level. *B. napus* is a valuable oil crop and an ideal polyploid model for studying the evolution, domestication and genetics of polyploids. However, a systematical investigation of the *BnUBC* family *s* has not been conducted to date. In this study, the availability of the *B. napus* whole-genome sequence provides opportunities for characterizing the *BnUBC* family and revealing its genetic effects on agronomic traits.

In this study, 200 *UBC* genes including two RUB genes, ten SUMO and 31 *UEV* genes, encoding UBC domain-containing ubiquitin-conjugating enzymes were detected in *B. napus*. The number of *UBC* gene in *A. thaliana*, maize, tomato, rice and *B. napus* (48, 75, 59, 39 and 200, respectively) are not correlated with the genome size (~125, ~466, ~2300, ~900, and ~825 Mb, respectively). Increasing evidence suggest that segmental duplications may be the main factor responsible for the expansion of gene families in plants (Wang et al., 2015; Liu et al., 2017; Wang et al., 2017). The loss and gain of genes during the process of polyploidization determine the genetic variability, which ultimately affects the function of the protein (Albalat and Cañestro, 2016). Analysis of gene structures and motifs of *BnUBC*s revealed that variation in the number of exons and introns as well as motif constitution may lead to their functional diversity.

*Cis*-acting elements in promoter regions regulate the transcription of genes that participate in various physiological processes (responses to hormone stress, plant growth and development) and that form the basic functional links among the complex regulatory networks (Nuruzzaman et al., 2014). Abundant *cis*-acting elements which were light-responsive and related to the hormone, plant growth and development were detected in the promoter regions of *BnUBC*s. Two *cis*-acting elements (TATA-box and CAAT-box) were presented in most *BnUBC*s (**Supplementary Figure 2**). Some *cis*-acting elements widely distributed in the eukaryotes, such as TATA-box and CAAT-box were also found in *BnUBCs*. These *cis*-acting elements constitute the RNA transcription factors binding sites (Laloum et al., 2013) and regulate the gene transcription process in conjunction with its binding factor. Moreover, other *cis*-acting elements such as MYB, MYC, ARE and ABRE, which are related to plant growth and development, were also found in the majority of *BnUBC*s. For example, MYB transcription factors function in plant breeding and response to stresses (Li et al., 2019) and MYC proteins function as transcriptional activators in abscisic acid (ABA) signaling (Abe et al., 2003). Besides, the abscisic-responsive element (ARE) *cis*-acting element is necessary for the anaerobic induction and the ABA-responsive element (ABRE) *cis*-acting element functioned in the response to ABA stress (Narusaka et al., 2003). Furthermore, many *cis*-acting elements involved in response to light were found in the *BnUBC* promoter regions, suggesting their potential role in regulating the pathways associated with light responsiveness. Overall, the *cis*-acting elements detected in *BnUBC* promoters suggested that they played crucial roles in plant growth, development and responses to abiotic stress and light.

Phylogenetic analysis divided BnUBC proteins into 18 groups, which is similar to the previous studies (Kraft et al., 2005; Dong et al., 2016; Sharma and Bhatt, 2017). In addition, the UBC domains showed uniform distribution in all the *UBC*s and gene expansion caused the variation of this core element during the evolution. Group XVIII possessed the largest number of *BnUBC*s with the most variation in gene structure and motifs, indicating that members in this group evolved more diverse functions than the other groups. Moreover, the phylogenetic classification of *BnUBCs* was further supported by the analyses of gene architecture and conserved motifs. Additionally, most of the members in the same subgroup shared similar gene structures and conserved motifs, but showed obvious different in *cis*-acting regulatory elements (**Supplementary Figure 2**), indicating their potential function diversity (Zou et al., 2011; Oudelaar and Higgs, 2021). Furthermore, the phylogenetic relationships of *AtUBC*s and *BnUBC*s suggested that gene duplication was likely a major factor in the diversification of *BnUBC* genes during evolution.

Gene duplication is considered a major mechanism leading to gene family expansion and functional diversification (Lynch and Conery, 2000; Prince and Pickett, 2002; Bianconi et al., 2018). In plants, three types of genome duplication have been reported, including WGD, tandem duplication and chromosomal segmental duplication (Ramsey and Schemske, 1998). According to many previous studies, WGD and segmental duplication are important factors for genome duplication and gene expansion (Ma et al., 2017; Wu et al., 2018; Zhu et al., 2020). After divergence from the ancestor of *Arabidopsis* lineage, *Brassica* species underwent WGT of approximately 13 MYA. Allotetraploid *B. napus* was formed by the interspecific hybridization between *B. rapa* and *B. oleracea* (Allender and King, 2010; Chalhoub et al., 2014). Thus, the genome size of *B. napus* was expanded and a single gene in *A. thaliana* corresponded to six copies in *B. napus* (Lysak et al., 2005). According to collinearity analysis, WGD showed a large contribution to genome expansion in *Brassica* species, followed by transposed duplication (**Table 1**). In this study, the number of *BnUBC*s (200) was approximately 4-fold higher than that of *AtUBC*s, while the number of *BraUBC* and *BolUBC* genes was <3-fold higher than that of *AtUBC*s, indicating gene loss in Brassica species during evolution (Albalat and Cañestro, 2016). Moreover, the numbers of *BnUBCs* in the A_n_ and C_n_ subgenomes were 97 and 103, respectively, which was almost consistent with that in the genomes of its progenitors *B. rapa* and *B. oleracea* genomes. These results demonstrate that most gene loss of *UBC*s occurred after whole genome triplication in *Brassica* ancestors and *BnUBC*s were mainly inherited from their progenitors (Chalhoub et al., 2014). Notably, several orthologs of *AtUBCs* (*AT3G57870*, *AT1G14400*, *AT5G56150*, *AT1G23260*, *AT1G17280*, *AT2G36060* and *AT3G08690*) maintained six copies in *B. napus* and were not loss after WGD, implying their crucial roles in plant growth and development. In *B. napus*, the Ka/Ks ratios between *UBC* paralogs were obviously less than one, indicating that these genes were undergone purifying selection during evolution.

The majority of *BnUBCs* localized to the nucleus, and only a few localized to the cytoplasm (**Supplementary Table 1**). This is consistent with a previous study, which showed their existence in the nuclear region for their involvement in fruit ripening (Wang et al., 2014). Moreover, these findings were also consistent with the results of GO enrichment analysis (**Supplementary Figure 3**). GO enrichment analyses predicted that *BnUBC*s are mainly involved in ubiquitination, cell growth and response to stresses (**Supplementary Figure 3**, **Supplementary Table 11**). However, the functional analysis of *BnUBC*s had been lacking. Therefore, we analyzed the expression profiles of these *BnUBC*s in various tissues (bud, callus, root, silique, and leaf) to predict their possible functions. The RNA-seq data (Yao et al., 2020) showed that the expression levels of *BnUBC*s varied across various tissues, consistent with previous studies (Cui et al., 2012; Jeon et al., 2012; E et al., 2015; Jue et al., 2018). For instance, *AtUBC1* and *AtUBC2* expressed in root, leaf, flower and seedling (Xu et al., 2009) And their orthologs in *B. napus* showed relatively high expression in bud, root and silique. The average expression level of *BnUBCs* was low in the leaf and the least number of genes expressed in callus (**Figure 7**, **Supplementary Table 12**). Moreover, diverse expression patterns were found between several duplicated genes, suggesting their divergence through subfunctionalization, neofunctionalization or pseudogenization in *B. napus* polyploidization (Chaudhary et al., 2009). Moreover, the difference of *cis*-acting regulatory elements in the promoter regions between *BnUBC*s may contribute to their expression level and function divergences.

To reveal the genetic effects of *BnUBC*s on agronomic traits, SNPs of *BnUBC*s were detected in accessions comprising a *B. napus* natural population (Tang, 2019) (**Supplementary Table 13**). The average SNP density of Bn*UBC*s (17 SNPs/kb) was slightly higher than that of genes in the entire genome (13 SNPs/kb), suggesting that a large number of polymorphisms accumulated in *BnUBC*s during evolution. The SNP density of *BnUBC*s in the A_n_ subgenome was higher than that in the C_n_ subgenome, which is consistent with some other gene families in *B. napus* (Zhu et al., 2020; Wahid et al., 2022; Xie et al., 2022). Classically, the difference in genetic variations between paralogs may lead to subfunctionalization, pseudogenization or neofunctionalization (Schiessl et al., 2017). Several duplicated *BnUBC* gene pairs, such as *BnaA03g11080D*/*BnaC02g12440D* and B*naA07g03330D*/*BnaC03g46750D*, showed significant different in the number of SNPs. Differences were also observed in the expression levels of these duplicated genes, implying their functional divergence. Furthermore, association mapping analysis of *BnUBC*s, based on SNPs, revealed their genetic effects on agronomic traits. For example, *BnaC02g25260D* was significantly associated with plant height (**Figure 8**). According to the results of GO enrichment analysis, *BnaC02g25260D* -interacting protein were likely involved in the regulation of plant cell growth, hormone levels and chlorophyll biosynthetic process, and could eventually influence plant height (Wang X, et al., 2020; Dong et al., 2021a). In addition, *BnaAnng34240D* was significantly associated with agronomic traits such as flowering time and main inflorescence silique density (**Supplementary Figure 4**). Ubiquitination, as reported in previous studies, is one of the crucial mechanisms that control the photoperiodic regulation of floral organ development (Sadanandom et al., 2012; Piñeiro and Jarillo, 2013). Moreover, the interacting proteins of BnaAnng34240D were involved in protein modification, regulation of transcription and so on. *AtSUMO1/2* and S *AtSCE1a* (ortholog of *BnaAnng34240D*) are implicated in ABA responses and the ubiquitin-like SUMO protease 1 (ULP1) gene *AtESD4* is involved in flowering time regulation (Miura et al., 2007). In addition, *BnaA07g28330D* was significantly associated with TSW, an important trait affecting crop yield. The obviously different expression levels of *BnaA07g28330D* between high and low oil content accessions in silique, implying its effects on oil content. Thus, the results of this analysis in the present study provided a valuable resource including candidate *UBC* genes affecting agronomic traits in *B. napus*.

To date, the *UBC* family were reported in several plant species. In this study, the characteristics of the *BnUBC* were compared with those of the *UBC* family in other plant species to help broaden our understanding of the differences or similarities in E2s across species. When compared with other species, this family in *B. napus* was largest and contained most subgroups suggesting the complexity of the allotetraploid genome. Moreover, majority of the *UBC*s were derived from WGD in *B. napus* and *Vitis vinifera* (Gao et al., 2017), whereas by segmental duplication in rice (E et al., 2015), tomato (Sharma and Bhatt, 2017), maize (Jue et al., 2015) and banana (Dong et al., 2016). The physicochemical properties of BnUBCs were similar to those in potato (Liu et al., 2019), maize (Jue et al., 2015) and banana (Dong et al., 2016). Additionally, and the chromosomal locations of the *BnUBC*s predicted in this study were partially consistent with previous reports (Miura et al., 2011; Cui et al., 2012; Cheng et al., 2017).

Our study focused on the *UBC* family, which represents only a part of the UPS, which generally contains the sequential action of E1, E2, and E3 enzymes. To gain better insight into the regulatory functions of the UPS, we compared our results with the other family in UPS. WGD played a predominant role in the expansion of the ubiquitin family (Hua et al., 2018), which is consistent with *BnUBCs*. Different from the similarity of physicochemical properties within the *UBC* family across species, significant sequence diversities were discovered between Poaceae and Brassicaceae (Hua and Yu, 2019). Only two members of the *E1* family in *A. thaliana* were detected with experimental verification (Hatfield et al., 1997). In addition, these two genes did not exhibit different expression patterns and the encoded E1 proteins showed no significant difference in enzymatic activities. However, the *BnUBC*s differentially expressed in various tissues and displayed various gene structures and motifs between different subgroups. With regard to the *E3* family, it can be divided into three types according to their subunit composition and functional mechanism, including homologous to E6-AP carboxyl terminus (HECT), Really Interesting New Gene (RING) and U- box type and cullin-RING ligase (CRL) (Vierstra, 2009). Thus, the *E3* family was more complicated than the *UBC* family based on the classification of its domains. The U-box type *E3* family contains 79 members in maize (Li et al., 2022), 68 in sorghum (Fang et al., 2022) and 62 in tomato (Sharma and Taganna, 2020); this family is slightly larger in size than the *E2* family. Furthermore, the *E3* genes are involved in plant development, growth and abiotic stress response, which is consistent with the functions of *E2* genes reported previously (Sharma and Taganna, 2020; Wang et al., 2021; Fang et al., 2022; Li et al., 2022; Lin et al., 2022).

## Conclusion

In the present research, we conducted a comprehensive investigation of the *UBC* family in *B. napus*. A total of 200 *BnUBC*s were identified and classified into 18 groups. The gene structures and motifs were highly conserved among members of the same phylogenetic subgroup. Moreover, *cis*-acting elements found in the *BnUBC*s promoters and the expression patterns of *BnUBC*s in diverse tissues demonstrated that these genes play a crucial role in plant growth and development. In addition, synteny analysis of the *UBC*s between *B. napus* and three ancestors (*A. thaliana*, *B. oleracea* and *B. rapa*) revealed the expansion history of the *BnUBC* gene family. Furthermore, genetic variations identification and association mapping analyses of *BnUBC*s uncovered their potential genetic effects on agronomic traits related to oil content and yield in *B. napus*. Overall, this study provided useful information about *BnUBCs* and will facilitate functional studies as well as the genetic breeding of *B. napus* in the future.

## Data availability statement

The datasets presented in this study can be found in online repositories. The names of the repository/repositories and accession number(s) can be found in the article/**Supplementary Material**.

## Author contributions

SY, CT, and XY designed the research. SY, HW, XL and PH performed the experiments. SY, MX, MH and XC analyzed the data. SY, CT, and XY wrote and revised the manuscript. All authors contributed to the article and approved the submitted version.
