# Supplementary figures and images for: Genome-wide characterization of ubiquitin-conjugating enzyme gene family explores its genetic effects on the oil content and yield of Brassica napus
Source: Front Plant Sci. 2023 Mar 20;14:1118339. doi: 10.3389/fpls.2023.1118339 (PMC10067767; doi:10.3389/fpls.2023.1118339)

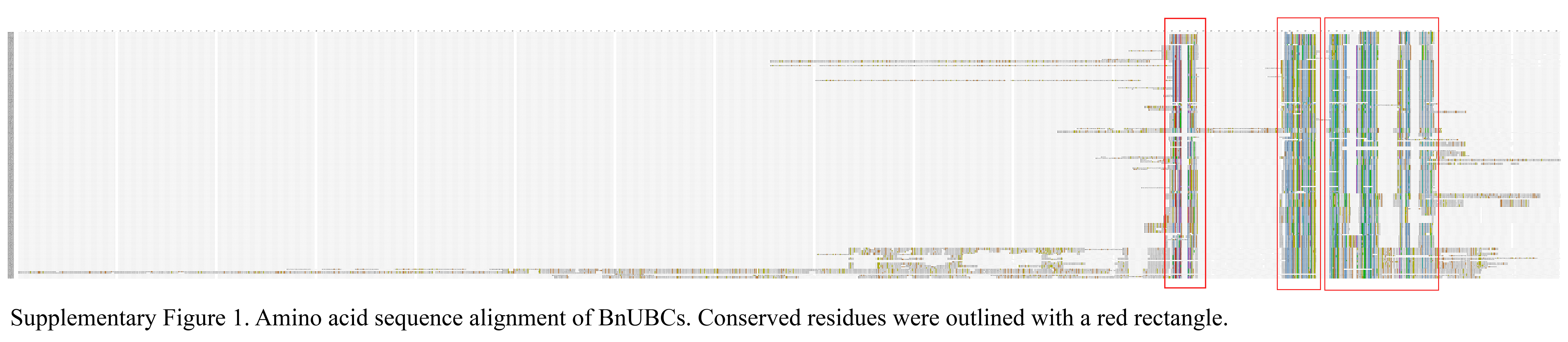

Supplement: Supplementary file 1 [file Image_1.jpeg]

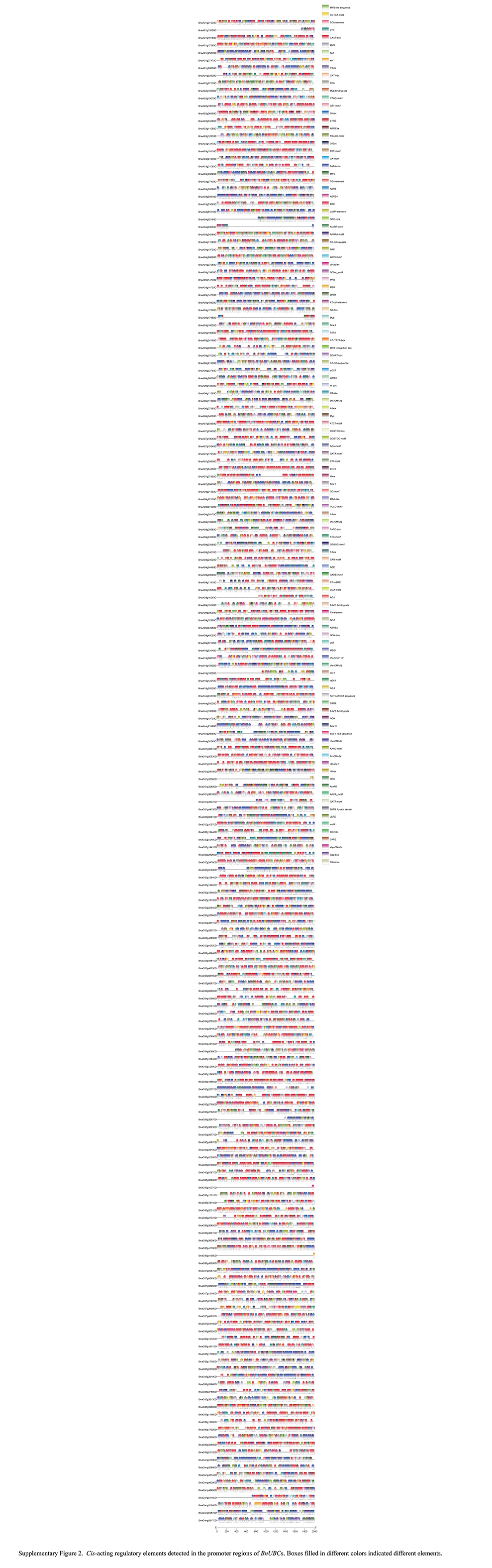

Supplement: Supplementary file 2 [file Image_2.jpeg]
